# Notes

**Published:** 1883-01

**Authors:** 


WE BEG to inform our readers that, in future, the JOURNAL will be published by William R. Jenkins, 850 Sixth Avenue, N. Y., well known as a publisher of Veterinary Works. It is requested that all communications relating to the JOURNAL will be forwarded to the Editor in care of Mr. Jenkins.

---

THANKS are returned to John Faust, V.S., Poughkeepsie, N. Y., for a fine specimen of Bovine Tuberculosis forwarded to the museum of the Columbia Veterinary College and School of Comparative Medicine.

